# ECS-tea: a bio-inspired high-precision detection and localization algorithm for young shoots of Pu-erh tea

**DOI:** 10.3389/fpls.2025.1697209

**Published:** 2025-12-03

**Authors:** Jianchao Wang, Wei Li, Jing Xu, Hailong Ti, Chenxi Jiang, Hongsen Liao, Jianlong Li, Quyun Li

**Affiliations:** School of Mechanical and Traffic Engineering, Southwest Forestry University, Kunming, China

**Keywords:** Pu-erh tea, YOLOPose, object detection, pose estimation, depth camera, smart agriculture

## Abstract

**Introduction:**

Pu-erh tea, valued for its ecological significance and economic worth, requires precise and efficient bud harvesting to advance intelligent agricultural operations. Accurate bud recognition and localization in complex natural environments remain critical challenges for automated harvesting systems.

**Methods:**

To address this, we propose ECS-Tea, a bio-inspired and lightweight detection-localization framework based on YOLOv11-Pose, tailored for Pu-erh tea bud analysis. The framework integrates four key modules: (1) a lightweight EfficientNetV2 backbone for efficient feature representation; (2) a Cross-Scale Feature Fusion (CSFF) module to strengthen multi-scale contextual information; (3) a Spatial-Channel Synergistic Attention (SCSA) mechanism for fine-grained keypoint feature modeling; and (4) an adaptive multi-frame depth fusion strategy to enhance 3D localization precision and robustness. ECS-Tea was trained and validated on a dedicated dataset for Pu-erh tea bud detection.

**Results:**

Experimental results show that ECS-Tea achieves 98.7% target detection accuracy and 95.3% keypoint detection accuracy, with a compact architecture (3.3 MB), low computational cost (4.5 GFLOPs), and high inference speed (370.4 FPS). Compared to the baseline YOLOv11-Pose, ECS-Tea significantly improves keypoint detection performance: mAP@0.5(K) increases by 4.9%, recall R(K) by 3.8%, and precision P(K) by 3.4%, while maintaining or slightly enhancing object detection metrics.

**Discussion:**

These findings demonstrate that ECS-Tea effectively balances accuracy and computational efficiency, validating the complementary contributions of its integrated modules. As a robust, real-time, and deployable solution, it bridges the gap between algorithmic sophistication and practical application, enabling high-precision tea bud harvesting in unstructured field environments.

## 1 Introduction

Pu-erh tea, as one of Yunnan Province’s most emblematic specialty agricultural resources, has earned a distinguished status in both domestic and international tea markets owing to its unique mellow flavor and profound cultural connotations (National Intellectual Property Administration of China, 2008; Jin et al., 2023).In recent years, the ancient-tree Pu-erh tea market has experienced sustained growth at an average annual rate of about 12%, intensifying the supply–demand imbalance for high-quality raw materials. During the harvesting stage, the accuracy of bud identification and spatial localization directly determines tea quality and economic yield. The traditional manual picking mode, which relies heavily on the experience of skilled tea farmers, yields a daily per capita picking volume below 15 kg. This process is labor-intensive, difficult to standardize, and consequently results in marked variability in product quality across batches (Tang et al., 2022). Furthermore, Pu-erh tea trees, being tall arbor species with widely dispersed branches, often require climbing or the use of auxiliary tools for harvesting, thereby heightening safety risks and reducing operational efficiency. The Pu-erh tea plantations are typically scattered within forested terrains, where wind-induced motion causes continuous bud oscillation, leading to highly unstable visual information—in stark contrast to the dwarf, densely planted, and imaging-stable environments characteristic of terrace-grown tea (Zhou et al., 2017).

Amid the vigorous advancement of smart agriculture, computer vision technology—owing to its non-contact and high-resolution sensing capabilities—has opened new avenues for intelligent tea harvesting. The YOLO algorithm family, distinguished by its end-to-end real-time detection architecture, has achieved notable success across multiple agricultural applications and is now exhibiting emerging potential within tea plantation environments (Li et al., 2023). However, the growth environment of Pu-erh tea is inherently complex and variable, characterized by interlaced branches and dense foliage, which create a highly cluttered and interference-prone visual background. Moreover, tender shoots differ markedly in size, orientation, and angular configuration—their bud-tip direction and leaf unfolding angle fluctuate substantially—posing significant challenges for stable recognition and precise structural modeling demanded by mechanical harvesting systems. In addition, the considerable height of tea trees, combined with frequent occlusion and overlap, further compounds the complexity of visual perception and recognition. Consequently, YOLO-based detectors are prone to missed detections and false positives when applied to small and visually ambiguous targets in such conditions. With the rapid proliferation of deep learning techniques in agricultural domains, tea bud detection has emerged as an area of growing research interest. For instance, Xu et al. introduced a dual-branch architecture combining YOLOv3 with DenseNet201, which effectively differentiates tea buds from stems and achieves high detection accuracy across multiple viewing angles (Xu et al., 2022). Shuai et al. developed the YOLO-Tea model, incorporating CARAFE and Bottleneck Transformer modules alongside a six-keypoint regression mechanism, thereby enabling spatial structural perception and precise extraction of tea bud picking points (Shuai et al., 2023). Zhu et al. integrated the YOLOv5 framework with 3D point cloud analysis, employing DBSCAN clustering to accomplish precise localization of picking points, and achieved high accuracy and real-time performance in unstructured tea plantation environments (Zhu et al., 2023). Zhang et al. introduced a lightweight detection model that integrates EfficientNetV2 with the Ghost module, achieving a mean Average Precision (AP) of 85.79% while substantially reducing parameter complexity (Zhang et al., 2024). Wang et al. developed the YOLOv7-DWS model, which achieves an effective balance between detection accuracy and computational efficiency, attaining 93.38% recognition accuracy for tea buds under natural illumination conditions (Wang et al., 2025). Shi et al. designed a lightweight neural network capable of robust operation in complex field environments, thereby validating the practical feasibility of lightweight architectures in agricultural visual perception tasks (Shi et al., 2024). Beyond the YOLO family, CenterNet represents a representative non-YOLO framework that formulates object centers as keypoints and jointly regresses bounding boxes and keypoint offsets through heatmap-based representations (Zhou et al., 2019). Although it delivers accurate spatial perception, its dense decoding structure imposes computational burdens, limiting its real-time performance in field environments. In related domains, Dong et al. proposed RSNet, a compact-align detection head embedded lightweight network for small-object detection in remote sensing imagery, achieving an excellent accuracy–efficiency balance on high-resolution datasets (Dong et al., 2025a). Similarly, Dong et al. developed an industrial device-aided lightweight network for real-time rail defect detection, highlighting the deployment feasibility of compact architectures under constrained edge-device conditions (Dong et al., 2025b). These studies further underscore the importance of lightweight and deployable architectures for real-time field applications, aligning closely with the design philosophy adopted in this work.

Beyond traditional object detection, keypoint detection and pose estimation are emerging as key frontiers in structured visual perception, driving a shift toward finer-grained spatial understanding. Liu et al. introduced a keypoint-based weed growth point detection framework, which demonstrates remarkable robustness under complex environmental backgrounds (Liu M. et al., 2025). Deng et al. developed a joint recognition framework for tomato fruits and picking points, achieving high-precision spatial localization through a customized YOLO-Pose variant (Deng et al., 2025). These studies collectively demonstrate that integrating object detection with structural keypoint modeling can effectively overcome the inherent limitations of conventional 2D bounding-box–based approaches. Concurrently, lightweight architectures and attention mechanisms—including EfficientNet, MobileNetV3, CBAM, SE, and SCSA modules—are widely adopted in agricultural embedded systems to achieve an optimal trade-off between accuracy and computational efficiency. Moreover, multi-frame fusion and temporal modeling approaches are increasingly utilized to enhance detection stability under dynamic and unstructured field conditions (Imam et al., 2025; Liu G. et al., 2025; Liu et al., 2023). However, few studies have explored the integration of lightweight attention mechanisms with keypoint detection for precise localization of dynamically moving, high-altitude tea buds, leaving a critical research gap in fine-grained perception for agricultural robotics.

From a broader perspective, Wang et al. highlighted in their comprehensive review of visual intelligence in the tea industry that most existing studies remain centered on tea leaf detection and grading, while lacking deep integration with three-dimensional localization and robotic harvesting systems (Wang H. et al., 2023). Cao et al. underscored, from a broader perspective on the development of agricultural visual perception, the necessity of deeply integrating multimodal sensing with structural modeling to enhance the robustness, adaptability, and generalization capacity of intelligent agricultural systems (Cao et al., 2025).

In summary, while current research has substantially advanced tea bud detection and agricultural visual perception, several critical limitations persist:

1. Insufficient structural perception capability when confronted with dynamic environmental disturbances;
2. Inadequate three-dimensional localization accuracy, restricting its applicability to robotic arm manipulation and control;
3. Difficulty achieving a balance between detection accuracy and computational efficiency under lightweight deployment constraints;
4. Most current studies focus on terrace tea detection, while research on tender bud recognition of tall arbor-type Pu-erh tea trees remains scarce.

To this end, this study proposes a detection and localization framework for Pu-erh tea tender buds built upon an enhanced YOLO-Pose architecture, with the following key contributions:

1. A bionic-structure-inspired keypoint annotation strategy is introduced, establishing an eight-point keypoint and object detection dataset that incorporates visibility-aware annotations to capture the ecological structure of Pu-erh tea buds.
2. A lightweight EfficientNetV2 backbone network is developed, integrated with a Cross-Scale Feature Fusion (CSFF) module and an SCSA attention mechanism, to strengthen multi-scale feature representation while preserving high computational efficiency.
3. An adaptive multi-frame fusion strategy is proposed to enable spatiotemporal structural modeling and fine-grained localization of critical bud regions, offering robust visual guidance for high-elevation robotic harvesting systems.

This study aims to overcome the visual perception bottlenecks inherent in the complex, unstructured environments of tall arbor-type Pu-erh tea plantations, distinct from terrace tea systems. It provides a theoretical foundation and technical roadmap for developing intelligent harvesting equipment, thereby promoting the transition of the traditional tea industry toward precision, automation, and intelligence.

## 2 Materials and methods

### 2.1 Development of the Pu-erh tea tender shoot dataset

Taking Pu-erh tea trees from the UNESCO World Heritage Site—Jingmai Mountain as the study object, this research faithfully simulated real-world harvesting scenarios during data acquisition. The camera height ranged primarily from 1.5 to 2.5 meters, while tea trees typically exceeded 2 meters in height. Pronounced wind-induced motion resulted in frequent leaf fluttering and occlusion, thereby establishing a realistic semantic environment conducive to subsequent multi-frame fusion and robust recognition (Yang, 2024). This work is dedicated to resolving the challenges of bud recognition and spatial localization in complex natural environments, thereby laying a solid foundation for intelligent and automated Pu-erh tea harvesting.

#### 2.1.1 Data collection

**Figure 1:**
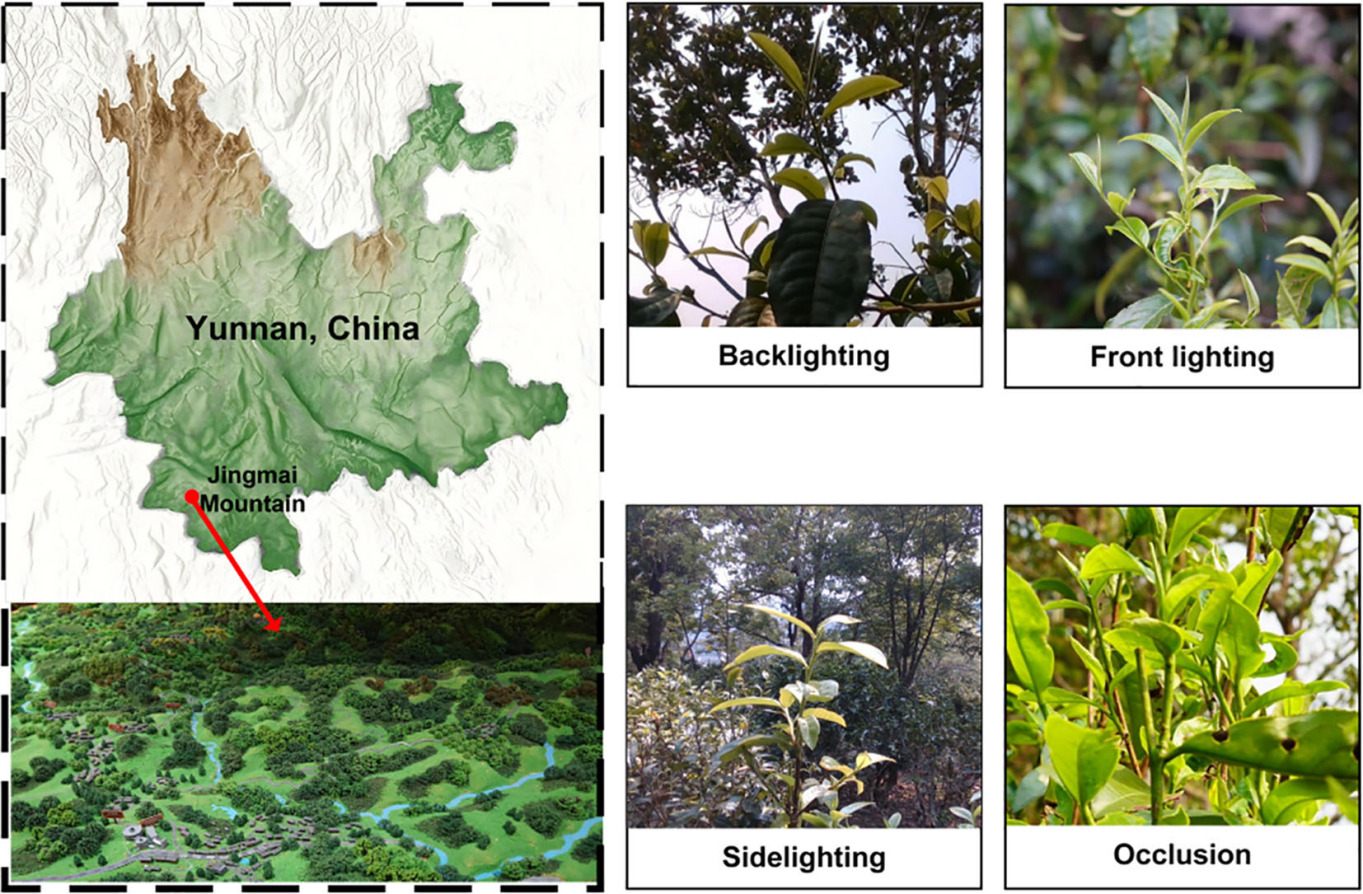
Original image dataset of Pu-erh tea tender buds.

Image data of Pu-erh tea buds were collected from tea plantations in Jingmai Mountain, Pu-erh, Yunnan Province (22°08′14″–22°13′32″ N, 99°59′14″–100°03′55″ E), at altitudes ranging from 1100 m to 1662 m. Using a high-resolution SONY Alpha 7III camera and an Intel RealSense D435i depth sensor, images were captured at distances of 0.3–2 m under diverse temporal, meteorological, illumination, and background conditions, as illustrated in **Figure 1**. Ultimately, a high-quality dataset comprising 1,769 curated images was constructed, serving as the foundation for model training and performance evaluation in subsequent experiments.

#### 2.1.2 Dataset augmentation and partitioning

**Figure 2:**
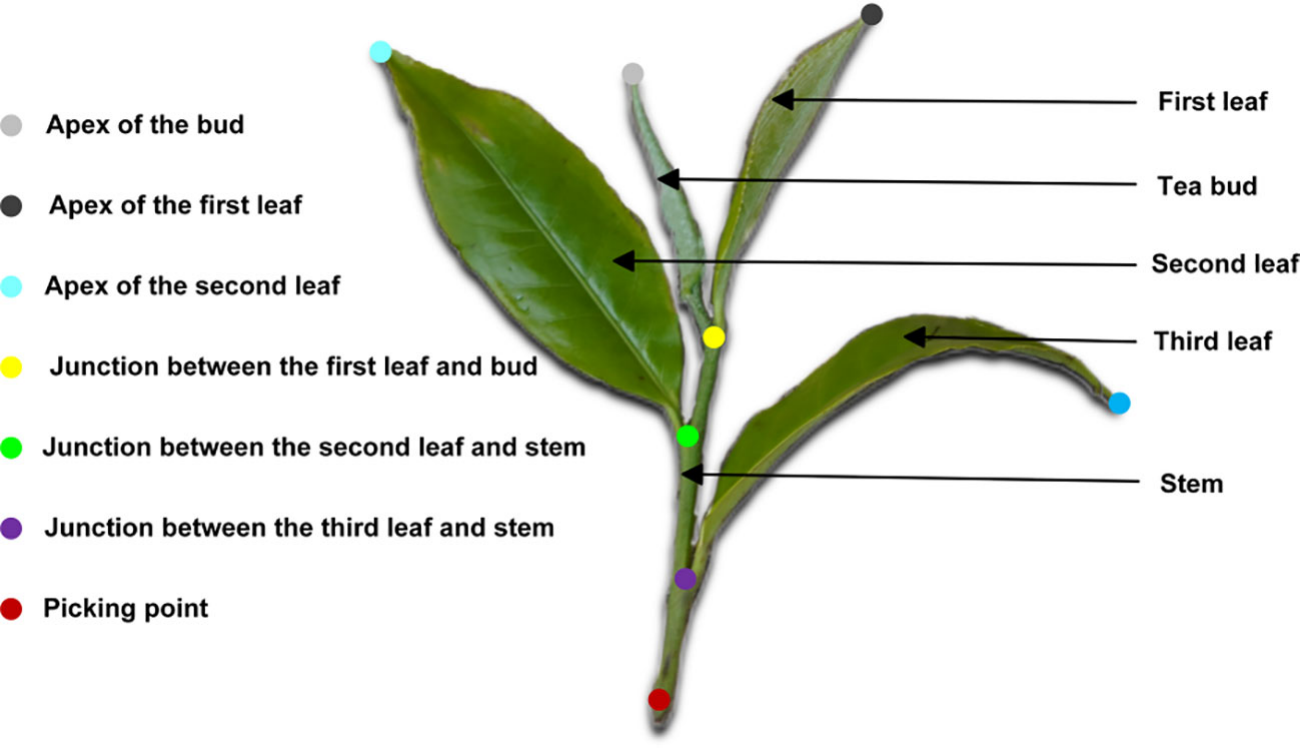
Illustration of keypoint annotation example.

In this study, Labelme was employed for image annotation. To enable fine-grained recognition and localization of Pu-erh tea buds, we adopted a bionic keypoint annotation strategy inspired by techniques in human pose estimation and animal structural analysis. The bud morphology was conceptualized as a human-analogous structure, with geometrically or functionally meaningful regions—such as the apex, base, and middle section—annotated as structural “key joints.” This human-inspired segmentation annotation approach allows the model to effectively learn the geometric topology and spatial constraints among the key structural components of the tea bud. After a thorough review of relevant literature and consultation with agronomic experts, the “one-bud–three-leaf” standard was adopted as the reference for harvesting, based on which the ecological keypoint locations for Pu-erh tea buds were precisely defined, as illustrated in **Figure 2**. Furthermore, visibility annotations were applied to the picking points: points partially occluded yet inferable from structural cues were marked as “occluded” (visible=1); points heavily occluded (>80%) and indeterminable were labeled as “invisible” (visible=0); while clearly discernible keypoints were designated as “visible” (visible=2). It is noteworthy that no internationally unified criterion exists for defining the picking point. In accordance with stem-harvesting requirements, this study defined the picking point as being 1–2 cm below the intersection between the third leaf and the main stem.

**Figure 3:**
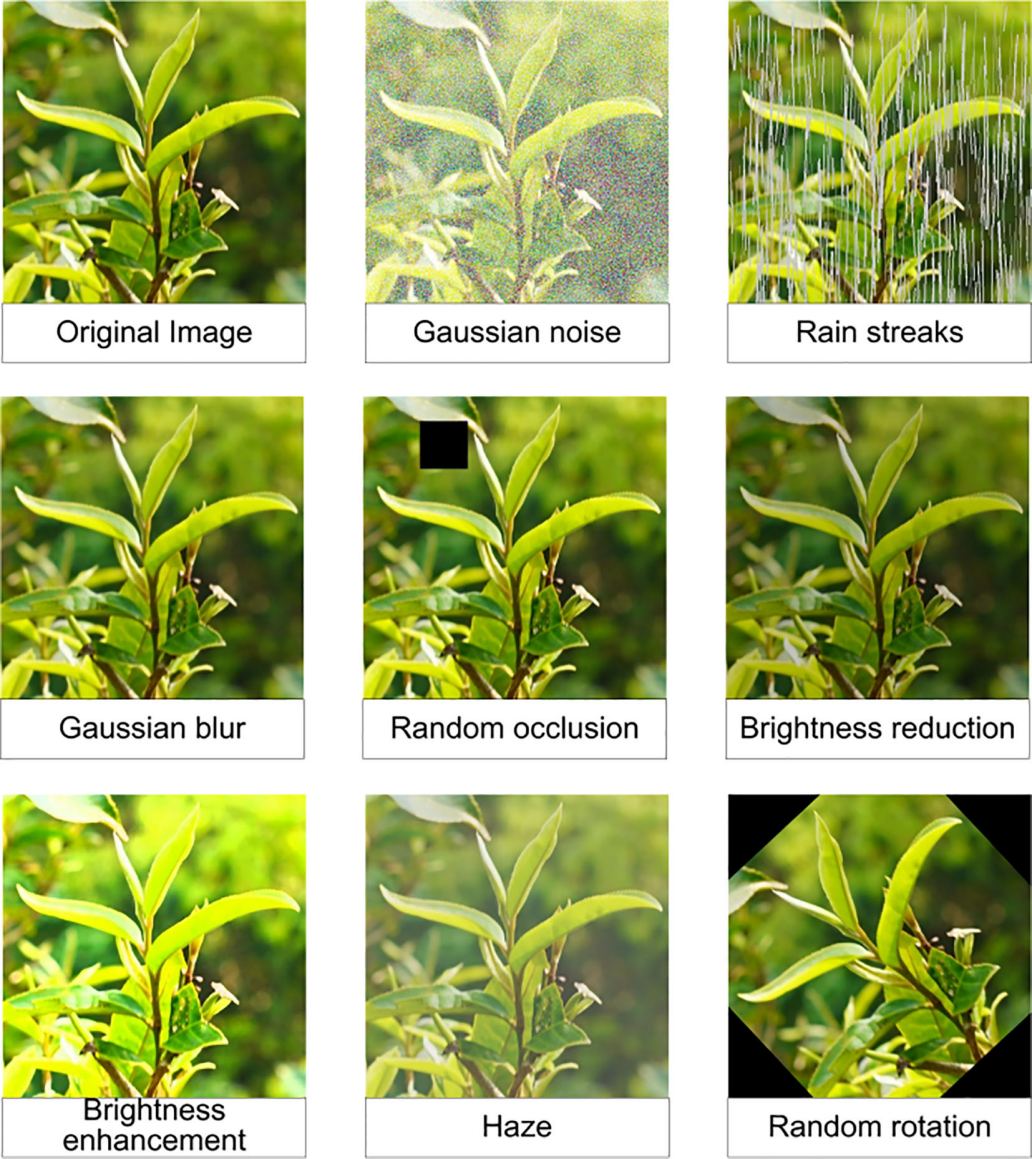
Enhancement method.

**Figure 4:**
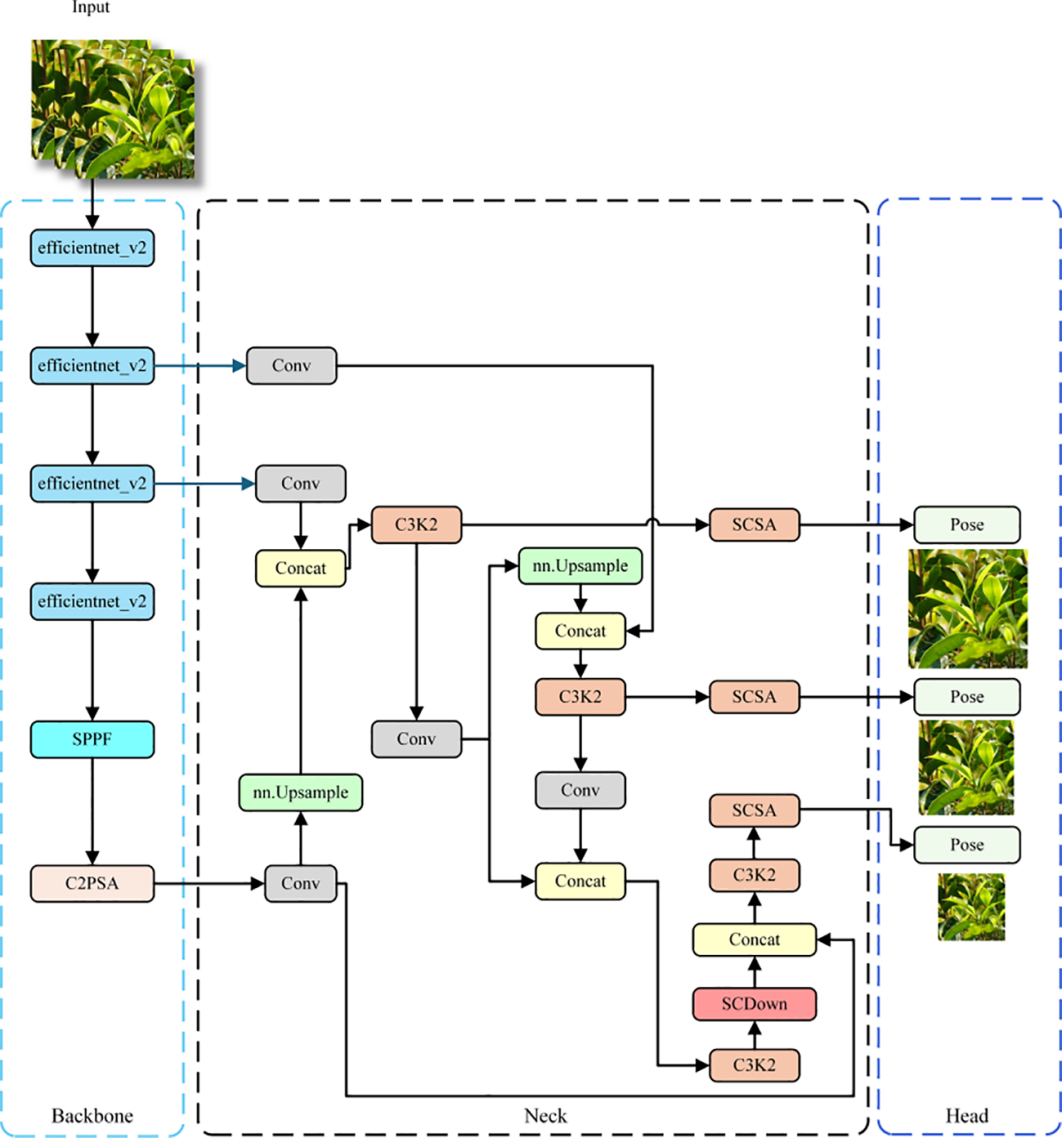
Schematic diagram of the ECS-Tea network architecture.

To improve the diversity and robustness of the Pu-erh tea bud image dataset and to address the limitations of insufficient samples and monotonous scene representation, this study integrated a comprehensive suite of image augmentation techniques (Bijelic et al., 2020; Shorten and Khoshgoftaar, 2019). Rain streaks, fog, and Gaussian noise were introduced to emulate complex environmental interferences, while brightness and exposure adjustments simulated diverse lighting conditions, as shown in **Figure 3**. Additionally, Gaussian blurring was applied to mimic imaging defocus, thereby enhancing the model’s resilience to real-world visual variability. Through rotation transformations and mirror flipping, the dataset was enriched with multi-perspective variations, strengthening the model’s capacity to recognize targets under diverse orientations and postures. Artificial occlusions were simulated by introducing black rectangular patches, which improved the model’s robustness and detection reliability under conditions of partial target obstruction. A label-synchronized transformation mechanism was further implemented to ensure perfect alignment between augmented images and their corresponding annotations. These augmentation strategies significantly expanded the dataset, furnishing a richer and more representative sample base for deep learning model training. This enhancement greatly improved the model’s generalization capability and stability in Pu-erh tea bud detection and recognition tasks. After augmentation, a total of 5,000 valid images were generated and randomly partitioned into training, testing, and validation subsets in a 7:2:1 ratio using Python scripts. **Figure 4** presents representative examples of images produced using the aforementioned augmentation techniques, illustrating the diversity and realism achieved through this process.

### 2.2 Model optimization strategies

YOLOv11, the latest generation in the YOLO family of lightweight real-time object detectors, achieves an exceptional balance between accuracy and computational efficiency, and has been extensively adopted across diverse intelligent vision applications (Khanam and Hussain, 2024). Building upon this framework, YOLOv11-Pose integrates an advanced keypoint regression module, allowing the network to perform not only bounding-box detection but also precise localization of internal structural keypoints, thereby extending its applicability to keypoint-intensive domains such as human pose estimation and agricultural structural analysis. Nevertheless, YOLOv11-Pose exhibits several limitations when applied to tea bud detection — its performance tends to degrade under complex backgrounds, low image quality, or drastic illumination changes, and it demonstrates limited robustness when dealing with small-scale or heavily occluded targets. To enhance its adaptability across diverse environmental conditions, this study proposes a structural optimization of YOLOv11-Pose, aimed at improving its recognition and localization capabilities for complexly structured tea buds, particularly within interference-prone and visually ambiguous Pu-erh tea harvesting environments. To overcome these limitations, this work introduces three major architectural enhancements: an EfficientNetV2 backbone for optimized feature encoding, a CSFF (Cross-Scale Feature Fusion) module for multi-scale feature enhancement, and an SCSA (Spatial–Channel Synergistic Attention) module for refined feature modeling. Collectively, these components constitute the ECS-Tea framework—a lightweight, high-efficiency, and robust detection network tailored for complex Pu-erh tea bud recognition.

Furthermore, the lightweight optimization not only enhances the computational efficiency of the model under resource-constrained environments, but also substantially reduces computational overhead and inference latency, thereby enabling seamless deployment on edge devices—such as tea-picking robotic manipulators—to ensure rapid and stable bud detection and localization in real-world harvesting scenarios (H.-I. Liu et al., 2024).

#### 2.2.1 Refinement of the overall network framework

This study presents a systematic optimization of the YOLOv11Pose architecture, as illustrated in **Figure 4**. EfficientNetV2 was adopted as a lightweight backbone. Leveraging its compound scaling strategy and enhanced MB Conv module, the network achieves substantial reductions in parameters and computational load, while maintaining a strong capacity for capturing essential features of Pu-erh tender shoots (Tan and Le, 2021; Wu et al., 2019).

The network incorporates the CSFF (Cross-Scale Feature Fusion Module), enabling multi-scale feature interaction and fusion (Zhao et al., 2024). This improves the network’s sensitivity to tender shoots and keypoints across scales and reduces the incidence of missed or false detections resulting from variability in shoot morphology. The SCSA (Spatial-Channel Split Attention) mechanism was also innovatively integrated, enabling refined feature selection and enhancement across both spatial and channel dimensions (Si et al., 2025). It guides the network’s focus toward salient features, thereby further enhancing detection accuracy for fine structural details and keypoints of tender shoots.

Collectively, these optimization strategies work synergistically to achieve a lightweight architecture while markedly enhancing the detection accuracy of Pu-erh tender shoots and their keypoints, thereby providing a reliable and accurate technical foundation for Pu-erh tea harvesting and quality assessment.

#### 2.2.2 Efficient NetV2

**Figure 5:**
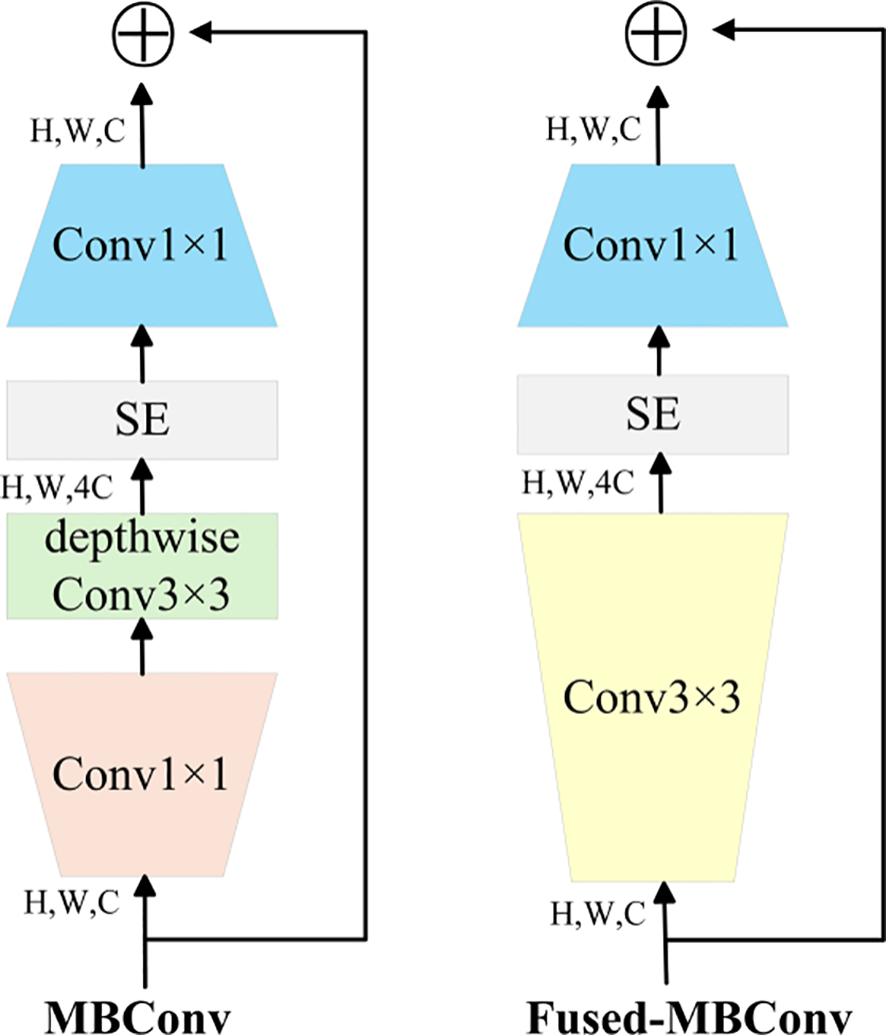
MB Conv and Fused-MBConv network structure.

EfficientNetV2 leverages training-aware Neural Architecture Search (NAS) and scaling strategies to optimize model performance. It was designed to overcome the training bottlenecks observed in EfficientNet (Tan & Le, 2020). In particular, it addresses inefficiencies in training large image sizes, low computational efficiency in deep convolutions within early layers, and the limitations of uniform scaling. Furthermore, progressive learning is applied during training. Regularization strength is adaptively scaled based on image size: initially, small images and weak regularization are used, while in later training phases, larger image sizes and stronger regularization are employed. The Fused-MB Conv structure is illustrated in **Figure 5** (Li et al., 2021). The architecture of EfficientNetV2 is detailed in **Table 1**.

**Table 1:** EfficientNetV2 network structure.

Stage	Operator	Stride	#Channels	#Layers
0	Conv3x3	2	24	1
1	Fused-MBConv1,k3x3	1	24	2
2	Fused-MBConv4,k3x3	2	48	4
3	Fused-MBConv4,k3x3	2	64	4
4	MBConv4,k3x3,SE0.25	2	128	6
5	MBConv6,k3x3,SE0.25	1	160	9
6	MBConv6,k3x3,SE0.25	2	256	15
7	Conv1x1&Pooling&FC	–	1280	1

In the context of tea bud detection, EfficientNetV2—owing to its highly efficient feature extraction and accelerated training performance—enables more precise and discriminative representation of bud features, thereby enhancing both detection accuracy and computational efficiency. Its compact parameter scale further minimizes resource consumption, facilitating practical deployment in real-world agricultural environments and offering robust technical support for tea bud detection and related research endeavors.

#### 2.2.3 Cross-scale feature fusion

**Figure 6:**
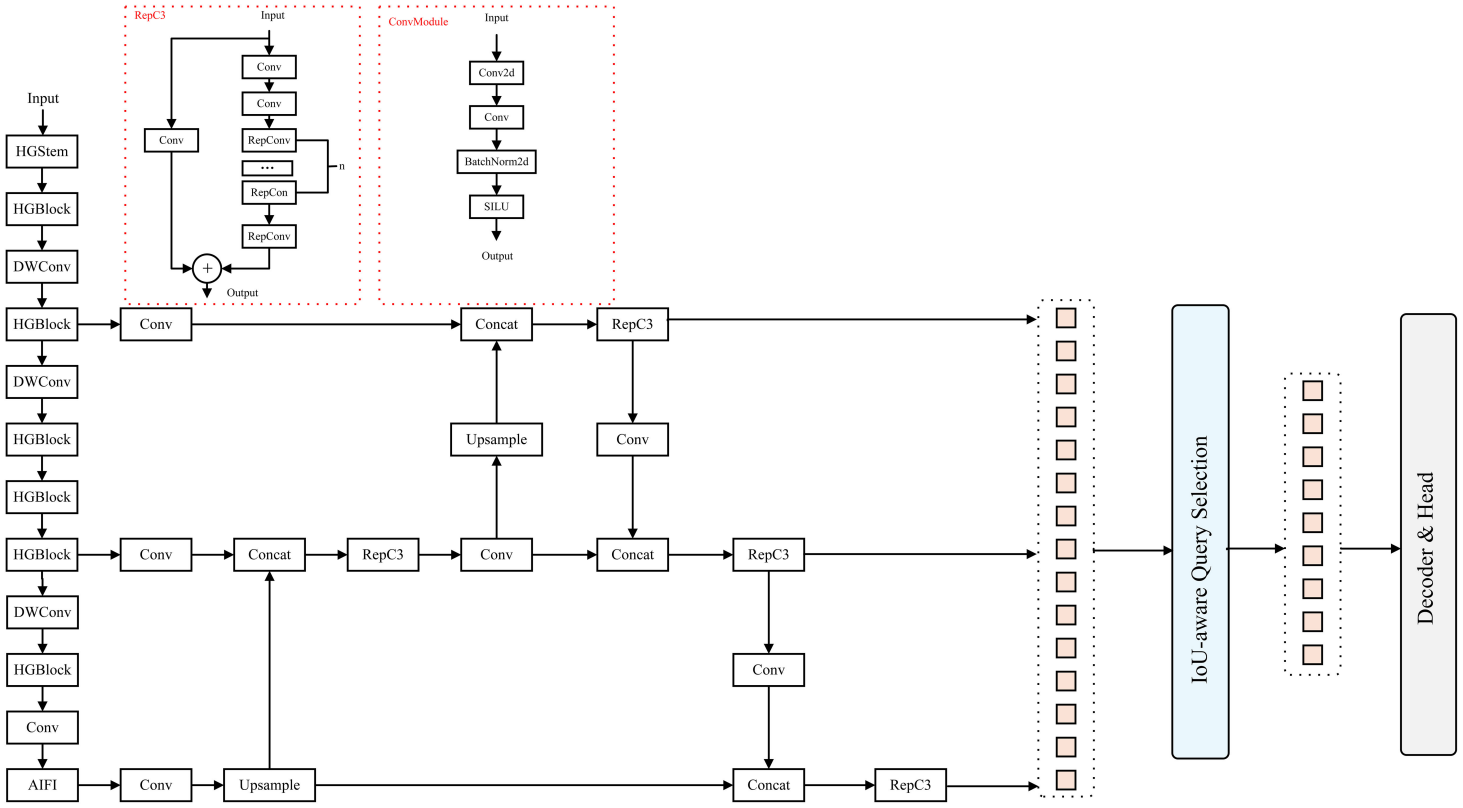
Structure of the CSFF module.

The CSFF (Cross-Scale Feature Fusion) module is designed to integrate multi-scale features, enhancing the model’s robustness to scale variations and improving small object detection. The structure is depicted in **Figure 6**. It builds upon and optimizes traditional cross-scale fusion modules through the incorporation of fusion blocks that integrate adjacent scale features. Each fusion block includes two 1×1 convolutions for channel adjustment, followed by N Rep Blocks to perform feature fusion, and computes the final fused feature through element-wise addition (Ding et al., 2021). This facilitates effective integration of multi-scale features.

In the overall architecture, the CSFF module is integrated into the neck section of the Pu-erh tea bud detection network. The multi-scale features extracted from the backbone are adaptively fused through the CSFF module, thereby strengthening semantic interactions and structural associations among different scales. Conventional neck fusion methods (e.g., FPN, PANet, BiFPN) generally rely on fixed topological connections or static weighting for feature propagation, often leading to insufficient feature alignment and semantic information loss. In contrast, the CSFF module adopts a content-aware cross-scale fusion strategy that adaptively models semantic dependencies across multiple feature scales, allowing high-level semantic cues to effectively complement low-level fine-grained details. This design maintains feature consistency and discriminative capability when handling tea bud targets exhibiting substantial scale variations.

The CSFF module is integrated into the neck of the tea detection architecture. Multi-scale features from the backbone are passed into the CSFF module, which leverages its fusion capability to enhance inter-scale information exchange. Conventional neck structures often suffer from information loss or inadequate feature fusion when processing multi-scale inputs, whereas the incorporation of CSFF effectively addresses these limitations. It enables the model to effectively capture tender shoot characteristics at various scales, thereby enhancing the feature representation available for downstream detection and localization.

#### 2.2.4 Spatial and channel synergistic attention

**Figure 7:**
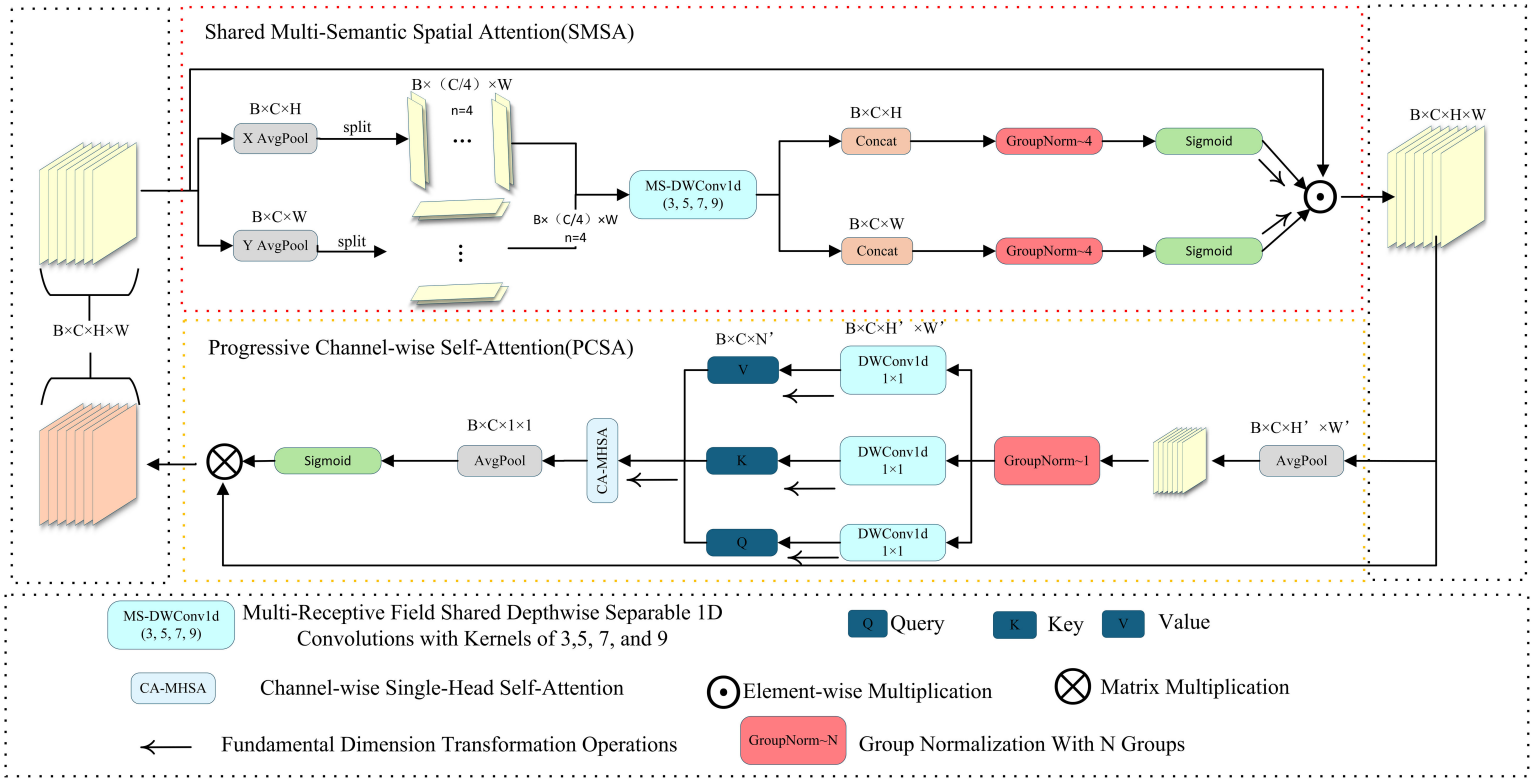
Structure diagram of SCSA.

The SCSA module draws inspiration from CBAM and CPCA, yet introduces targeted innovations in the mechanisms of spatial–channel interaction and feature fusion to better suit the requirements of fine-grained perception (Woo et al., 2018; Huang et al., 2024).Structurally, it comprises two components—Shared Multi-Semantic Spatial Attention (SMSA) and Progressive Channel-wise Self-Attention (PCSA)—whose overall architecture is depicted in **Figure 7**. Unlike conventional serial attention schemes, SCSA employs a bidirectional spatial–channel collaborative modeling strategy that jointly optimizes spatial and channel representations, thereby preserving salient feature information while effectively suppressing background noise.

In the spatial branch, SMSA generates the spatial attention map by combining spatial–channel decomposition with multi-receptive-field depth wise separable convolutions (kernel sizes 3, 5, 7, 9), Group Normalization, and Sigmoid activation, enabling multi-scale spatial context modeling. The multi-branch convolution design allows the model to capture spatial cues across multiple receptive fields, facilitating the accurate recognition of slender Pu-erh tea shoots even under challenging conditions such as cluttered backgrounds and uneven illumination. Unlike the conventional CBAM, SMSA integrates a shared multi-semantic extraction strategy within its spatial attention module, enabling semantic complementarity across branches and thus strengthening multi-scale spatial representation. In the channel branch, PCSA integrates a single-head self-attention mechanism with a progressive channel compression strategy, progressively modeling inter-channel dependencies to emphasize semantically salient features while suppressing redundant activations. The SMSA and PCSA modules are integrated via a residual pathway (**Figure 7**), wherein the spatial attention output reciprocally guides channel attention learning, establishing a closed-loop feature collaboration mechanism that synchronously optimizes spatial and channel representations. This collaborative attention design effectively overcomes the limitations of traditional attention modules (e.g., SE, CBAM), which rely on a fixed sequential order of spatial and channel processing, thereby endowing the model with stronger adaptability and generalization in structured feature extraction. Within the overall network architecture, the SCSA module is embedded in the neck section, as shown in **Figure 4**. Specifically, it is positioned after each multi-scale fusion block (C3K2) and before the detection and keypoint heads, where it jointly enhances the fused features across the three hierarchical levels (P3, P4, P5) through spatial–channel cooperation. This design refines salient region representations following multi-scale feature fusion, enabling the network to more precisely perceive and localize the key structural regions of Pu-erh tea buds.

Furthermore, to achieve a balance between performance and efficiency, the SCSA module employs a lightweight depth wise separable convolution design, which maintains low computational overhead while surpassing existing attention and multi-scale fusion frameworks (e.g., SE, CBAM, BiFPN) in terms of detection accuracy and feature stability for Pu-erh tea bud recognition, thereby validating its structural innovation and strong task adaptability.

### 2.3 Deep positioning analysis

Considering the challenges of unstable viewpoints, structural ambiguity, and keypoint jitter during Pu-erh tea harvesting, this study introduces an adaptive multi-frame depth fusion module built upon the 2D picking-point outputs of the ECS-Tea model. By integrating keypoint information across consecutive frames, the method enables dynamic and stable spatial modeling of tea buds, effectively mitigating the accuracy degradation encountered by conventional detection methods under high-elevation vibration conditions. The approach employs an Intel RealSense D435 depth camera to perform continuous-frame depth sampling of identical picking points and applies an adaptive multi-frame fusion strategy to refine depth estimation, thereby substantially enhancing the stability and accuracy of 3D spatial coordinates.

#### 2.3.1 Inter-frame keypoint tracking mechanism

To guarantee that depth samples across multiple frames correspond to the same physical location within the image, an inter-frame keypoint tracking mechanism is incorporated. In the absence of such a mechanism, camera shake, subtle movements, or detection biases may cause slight shifts in keypoint positions, thereby compromising the spatial consistency of depth fusion. The keypoint tracking procedure is outlined as follows:

1. Initial detection: In frame t, the ECS-Tea model extracts the 2D pixel coordinates (u_t_, v_t_) of keypoints.
2. Keypoint matching: To enhance the robustness and accuracy of inter-frame keypoint tracking, this study adopts the Transformer-based LoFTR (Detector-Free Local Feature Matching) algorithm, replacing conventional Lucas-Kanade optical flow methods. Across consecutive frames, LoFTR applies an end-to-end trained Transformer to encode global image features, thereby facilitating cross-frame feature matching, and does so without relying on local window constraints or grayscale consistency assumptions (Sun et al., 2021). For each keypoint detected in the current frame, LoFTR autonomously locates the globally optimal match in the subsequent frame, establishing inter-frame correspondences and yielding a sequence of keypoint coordinates over N consecutive frames. In this work, the fusion frame count (N) was empirically set within the range of 3–10 frames, a configuration derived from the hardware specifications of the Intel RealSense D435i depth sensor integrated into the experimental platform. Given the sensor’s depth sampling rate of 90 fps, a 3–10 frame temporal window provides an optimal trade-off between spatial coherence and real-time computational performance. When N < 3, depth estimation becomes insufficiently smoothed; conversely, when N > 10, latency and temporal jitter errors grow substantially, degrading overall system stability. A comprehensive evaluation indicates that, at the operational frame rate of the Intel RealSense D435i, a 3–10 frame fusion window achieves the optimal equilibrium between depth-fusion stability and real-time responsiveness.

$$\left({\text{u}}_{\text{t}},{\text{v}}_{\text{t}}),({\text{u}}_{\text{t}+1},{\text{v}}_{\text{t}+1}),\ldots ,({\text{u}}_{\text{t}+\text{N}-1},{\text{v}}_{\text{t}+\text{N}-1}\right)$$

3. Consistency check: For each tracked keypoint position, calculate the Euclidean distance Δ_i_ between the keypoint position and its corresponding position in the previous frame, The specific expression is shown in Equation 1:

(1) $${\text{Δ}}_{\text{i}}=\sqrt{{\left({\text{u}}_{\text{t}+\text{i}}-{\text{u}}_{\text{t}+\text{i}-1}\right)}^{2}+{\left({\text{v}}_{\text{t}+\text{i}}-{\text{v}}_{\text{t}+\text{i}-1}\right)}^{2}}$$

If Δ_i_ exceeds a predefined threshold ϵ (3 pixels), where the threshold selection is guided by empirical practices reported in (Sun et al., 2021) and (Teed and Deng, 2021) the tracking is deemed unsuccessful and the corresponding frame’s depth value is discarded. Keypoint tracking stability is typically evaluated over 2–3 frames. Considering the system’s resolution and practical matching accuracy, the threshold was set to 3 pixels.

Additionally, abnormal jitter frames are further filtered by analyzing temporal stability trends, thereby avoiding errors caused by motion blur or similar artifacts. This inter-frame tracking strategy ensures high spatial consistency of keypoints across frames, establishing a solid foundation for robust depth value fusion.

#### 2.3.2 Adaptive multi-frame depth fusion and 3D inverse projection

As raw images captured by depth sensors exhibit noise, holes, occlusions, and other artifacts across frames, single-frame depth measurements d often show substantial fluctuation. To enhance depth estimation accuracy, this study applies adaptive statistical fusion to the sequence of depth samples {d_1_, d_2_,…, d_n_} at corresponding keypoint positions across N frames and derives a more stable depth estimate d. The adaptive fusion strategy is defined as follows:

Standard deviation calculation: compute the standard deviation σ of the depth sequence to evaluate the stability of the depth measurements.

Fusion strategy selection: if σ≤T (threshold), the depth values are deemed stable, and mean filtering is employed. The threshold selection follows empirical values commonly adopted in recent depth estimation and dense reconstruction literature and is calibrated based on the sensor’s noise characteristics and the practical deployment scenario. The threshold is set to 0.01 m (Barron et al., 2022; Wei et al., 2021).

The specific expression is shown in Equation 2:

(2) $$\overset{\wedge }{d}=\frac{1}{M}\sum_{i=1}^{N}d_{i},d_{i}>0,M=\text{available frame count}$$

If σ>T, this indicates the presence of outliers or abrupt variations, in which case median filtering is applied to enhance robustness. The specific expression is shown in Equation 3:

(3) $$\overset{\wedge }{d}=median\{(d_{i}|d_{i}>0)\}$$

The fused stable depth value, along with 2D pixel coordinates (u,v), is input to the rs2_deproject_pixel_to_point inverse projection function provided by the Intel RealSense D435 camera, with spatial transformation performed based on the camera’s intrinsic parameters, yielding the final 3D coordinates (X,Y,Z). The specific expression is shown in Equation 4.

(4) $$\left(X,Y,Z)=rs2\_deproject\_pixel\_to\_point(K,(u,v),\overset{\wedge }{d}\right)$$

where K denotes the camera intrinsic matrix, comprising focal length, principal point coordinates, and additional parameters.

### 2.4 Experimental procedure

#### 2.4.1 Experimental environment

In this study, the software and hardware environments and their corresponding parameters are summarized in **Table 2**.

**Table 2:** Software and hardware environment.

Hardware/Software environment	Model/Identification	Parameter/Version
CPU	Intel Core i7-14900F	2.10 GHz
GPU	NVIDIA GeForce RTX 4060	6 GB
Operating System	Windows 11	22631.4460
Deep Learning Framework	PyTorch	1.12.1
Computing Platform	CUDA	12.1
Integrated Development Environment (IDE)	PyCharm	2024.1.3
Programming Language	Python	3.9.7

#### 2.4.2 Training parameters

The training parameters used in this study are listed in **Table 3**.

**Table 3:** Training parameters.

Batch size	Number of epochs	Initial learning rate	Final learning rate	Optimizer	Number of data loader workers
16	150	0.01	0.0001	SGD	4

The experimental settings were configured as follows: all input images were resized to 640×640 using the LetterBox method; the batch size was set to 16 during training and 1 during inference; automatic mixed precision (AMP) was enabled to enhance computational efficiency. All experiments were implemented in the PyTorch framework, without employing TensorRT acceleration. Data preprocessing followed the default YOLOv11 pipeline, comprising Mosaic augmentation, random perspective transformation, CopyPaste augmentation, MixUp blending, RandomHSV perturbation, and RandomFlip operations, followed by normalization and channel rearrangement. The latency and FPS values reported herein correspond exclusively to the detection and keypoint estimation stages, excluding the time consumed by LoFTR-based feature matching and fusion. These parameters were selected based on preliminary experiments to ensure stable convergence and optimal detection performance.

#### 2.4.3 Evaluation metric

This study adopts ten evaluation metrics to comprehensively assess the overall performance of the enhanced ECS-Tea model in both tea bud detection and keypoint localization tasks (Wang et al., 2022; Wang et al., 2022). These include object detection precision (P(B)) and keypoint precision (P(K)), object recall (R(K)) and keypoint recall (R(K)), as well as the average precision (mAP@0.5(K), mAP@0.5(B)) computed at an IoU threshold of 0.5. Additional efficiency metrics—parameter count, model weight size, floating-point operations (FLOPs), and frames per second (FPS)—were also analyzed to evaluate computational performance. The formulations for precision, recall, and average precision (AP) are expressed as shown in Equations 5–9:

(5) $$P=\frac{TP}{TP+FP}\times 100\%$$

(6) $$P=\frac{TP}{TP+FN}\times 100\%$$

(7) $$AP=\int_{0}^{1}P(R)dR$$

To achieve a more comprehensive assessment of the 3D keypoint localization accuracy of the proposed model, this study incorporates two complementary evaluation metrics—PCK (Percentage of Correct Keypoints) and OKS (Object Keypoint Similarity).

The PCK metric quantifies the proportion of predicted keypoints whose Euclidean distance from their corresponding ground-truth locations falls within a specified tolerance threshold (t), mathematically defined as shown in Equation 8:

(8) $$PCK@t=\frac{1}{N}\sum_{i=1}^{N}\text{δ}(d_{i}<t)$$

In this expression, N is the total number of keypoints, d_i_ refers to the three-dimensional prediction error of the i-th keypoint, and δ(·) denotes the indicator function. If the prediction error d_i_ is less than the threshold t, the point is classified as a correctly predicted keypoint.

The Object Keypoint Similarity (OKS) serves as a soft evaluation metric that incorporates target scale and prediction error, as shown in Equation 9:

(9) $$OKS_{i}=\exp(-\frac{d_{i}^{2}}{2S_{i}^{2}K^{2}})$$

In this context, d_i_ is the Euclidean distance between the predicted and true points, s_i_ designates the object scale (measured in millimeters), and k refers to the scale-adjusting factor. The closer the OKS value is to 1, the more accurate the prediction.

## 3 Results and discussion

### 3.1 Pu-erh tea tender shoot detection experiment

To thoroughly examine the effectiveness of the model architecture and algorithms, demonstrate the advantages of the proposed method, and intuitively assess detection performance, a series of ablation studies, comparative experiments, and visualization analyses were conducted. The ablation studies systematically decomposed the model’s key components in order to elucidate the practical contribution of each component to Pu-erh tea tender shoot detection performance. Comparative experiments were employed to conduct a cross-method comparison of various algorithms with the proposed approach, highlighting the superiority of the proposed method in terms of detection accuracy and efficiency. Visualization analysis was used to visually illustrate both the model’s detection process and its outcomes, for accurate diagnosis of detection error sources and thereby support subsequent model optimization. All experiments were performed under a consistent experimental environment and dataset.

#### 3.1.1 Model ablation study

YOLOv11Pose (denoted as A), EfficientNetV2 (module B), CSFF module (module C), and SCSA module (module D) were evaluated in the ablation study. The final experimental results, obtained under identical training, validation, and test conditions, are presented in **Table 4**.

**Table 4:** Ablation results.

A	B	C	D	P(B) (%)	R(B) (%)	mAP@0.5(B) (%)	P(K) (%)	R(K) (%)	mAP@0.5(K) (%)	FPS	Model size (MB)	Number of parameters	FLOPs(G)
✓				98.6	98.5	99.4	91.9	91.1	88.9	333.4	5.7	2696611	6.7
✓	✓			98.3	98.5	99.4	94.9	92.5	92.5	344.8	4.9	2201047	5.6
✓		✓		98.8	99.0	99.5	91.7	89.7	88.4	384.6	4.1	1859203	5.6
✓			✓	99.5	99.5	99.5	95.0	95.0	93.2	400.0	6.0	2834851	6.8
✓	✓	✓		99.0	98.8	99.4	93.2	93.0	90.5	357.1	3.2	1369271	4.5
✓	✓		✓	99.1	99.2	99.4	91.8	92.3	89.0	344.8	5.2	2339287	5.7
✓		✓	✓	98.9	99.4	99.5	93.3	93.8	90.9	357.1	4.1	1869187	5.6
✓	✓	✓	✓	98.7	99.2	99.5	95.3	94.9	93.8	370.4	3.3	1372343	4.5

The results indicate that, while maintaining object detection mean average precision (mAP@0.5(B)) consistently within the 98%–99% range, the model achieved a substantial improvement in keypoint detection mean average precision (mAP@0.5(K)), accompanied by a marked reduction in parameter count and computational complexity, thereby achieving an excellent balance between accuracy and efficiency.

With the introduction of EfficientNetV2, leveraging its compound scaling strategy and Fused-MBConv structure, the network achieved a more compact architecture and enhanced semantic modeling capability. The keypoint detection mean average precision (mAP@0.5(K)) increased from 88.9% to 92.5% (+3.6%), while the parameter count and floating-point operations were reduced by 18.3% and 1.1 GFLOPs, respectively—highlighting an optimal trade-off between performance and computational efficiency.

The Cross-Scale Feature Fusion (CSFF) module, through its cross-scale feature guidance mechanism, effectively enhances multi-scale feature integration. When applied independently, it achieved an mAP@0.5(K) of 91.7%, and when combined with EfficientNetV2, performance further improved to 93.2%, demonstrating a significant synergistic gain in multi-scale structural modeling.

The SCSA module introduces a spatial self-calibration mechanism, markedly amplifying keypoint-region responses and achieving an mAP@0.5(K) of 93.2%, the highest among all individual modules. Simultaneously, the inference speed increased to 400 FPS, reflecting exceptional localization sensitivity and real-time responsiveness.

Ultimately, integrating all three modules yielded the complete ECS-Tea model, which achieved an mAP@0.5(K) of 93.8%, representing a 4.9% improvement over the YOLOv11-Pose baseline. The keypoint detection accuracy (P(K)) increased from 91.9% to 95.3% (+3.4%). Concurrently, the parameter count dropped from 2,696,611 to 1,372,343 (–49.1%), FLOPs reduced from 6.7 to 4.5 GFLOPs (–32.8%), model weight decreased from 5.7 MB to 3.3 MB (–42.1%), and inference speed improved from 333.3 FPS to 370.4 FPS (+11.1%). These results confirm that ECS-Tea achieves a superior balance among accuracy, efficiency, and compactness, validating its potential for real-time deployment in resource-constrained agricultural systems.

#### 3.1.2 Model comparison experiment

To rigorously assess the effectiveness and superiority of the proposed ECS-Tea model, we conducted comparative experiments using identical datasets, augmentation strategies, and hyperparameter settings. The baselines comprised YOLOv5-pose, YOLOv7-pose, YOLOv8-pose, YOLO-Tea, and CenterNet, with ECS-Tea evaluated as the optimized reference model. YOLOv5-pose adopts a compact and robust architecture that exemplifies early efficient designs for joint object–keypoint detection. Building upon this, YOLOv7-pose and YOLOv8-pose improve localization accuracy and inference speed through enhanced feature aggregation and decoupled multi-scale detection heads. To enhance the completeness of comparison, we also incorporated YOLO-Tea—a model specifically designed for tea leaf detection—and CenterNet, a non-YOLO keypoint detection framework, serving as domain-specialized and cross-framework benchmarks.

**Figure 8:**
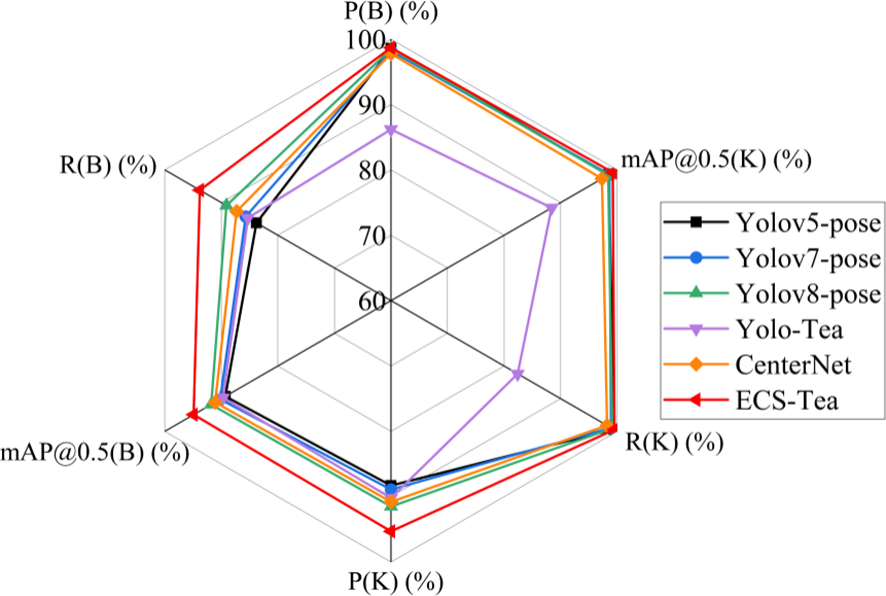
Comparative radar chart of object detection and keypoint detection performance across different models.

The models were comprehensively analyzed in terms of detection accuracy, computational complexity, and inference speed, as shown in **Table 5** and illustrated in **Figure 8**.

**Table 5:** Comparison of experimental results.

Model	P(B) (%)	R(B) (%)	mAP@0.5(B) (%)	P(K) (%)	R(K) (%)	mAP@0.5(K) (%)	FPS	Model size (MB)	Number of parameters	FLOPs (G)
Yolov5-pose	98.6	98.5	99.4	88.3	89.3	83.8	322.5	5.9	2770779	7.3
Yolov7-pose	98.2	98.6	98.9	88.9	90.2	85.7	330.8	6.3	4053675	8.2
Yolov8-pose	98.5	98.4	99.1	91.5	91.8	89.1	344.8	5.9	2798827	7.2
Yolo-Tea	86.2	88.4	82.4	90.2	89.7	85.3	342.7	16.9	3345263	12.8
CenterNet	97.8	97.3	98.2	90.8	91.1	87.3	235.6	15.8	5028341	10.9
ECS-Tea	98.7	99.2	99.5	95.3	94.9	93.8	370.4	3.3	1372343	4.5

In the object detection task, its mAP@0.5(B) reaches 99.5%, showing a slight improvement over YOLOv5-pose (99.4%) and YOLOv8-pose (99.1%), and a more significant gain compared with YOLOv7-pose (98.9%). This indicates that ECS-Tea possesses stronger feature representation capabilities under complex backgrounds and multi-scale target conditions. In the keypoint detection task, ECS-Tea achieves an mAP@0.5(K) of 93.8%, outperforming YOLOv5-pose (83.8%), YOLOv7-pose (85.7%), YOLOv8-pose (89.1%), and YOLO-Tea (85.3%) to varying degrees. Notably, it achieves the best keypoint precision (P(K)=95.3%) and recall (R(K)=94.9%).These results indicate that ECS-Tea exhibits better spatial consistency and feature alignment capability under high-interference scenarios.

As a representative non-YOLO framework, CenterNet achieves high accuracy in both object detection and keypoint localization (mAP@0.5(B) = 98.2%, mAP@0.5(K) = 87.3%), validating its regression-based localization capability. However, due to its dense heatmap decoding architecture, CenterNet incurs substantial computational overhead (model size: 15.8 MB, 10.9 GFLOPs, frame rate: 235.6 FPS), which limits its real-time performance and makes it unsuitable for embedded or field-deployment scenarios.

In terms of computational efficiency, the ECS-Tea model contains only 1.37M parameters, with a compact model size of 3.3 MB and a computation cost of 4.5 GFLOPs—substantially lower than YOLOv7-pose (8.2 GFLOPs) and YOLOv8-pose (7.2 GFLOPs). Its inference speed reaches 370.4 FPS, demonstrating remarkable real-time capability.

To intuitively illustrate the comprehensive performance of each model across multiple evaluation dimensions, **Figure 8** presents a multi-metric radar chart based on the data from **Table 5**. The results show that ECS-Tea forms the outermost envelope across detection accuracy (P(B), R(B), mAP@0.5(B)), keypoint localization (P(K), R(K), mAP@0.5(K)), and inference efficiency (FPS), indicating its superior overall balance among competing models. In contrast, YOLOv5-pose performs stably in object detection but shows lower keypoint accuracy due to its limited structural representation capacity. YOLOv8-pose demonstrates moderate improvements in certain metrics but suffers from higher computational cost and reduced real-time performance. YOLO-Tea captures features effectively for terrace-tea scenarios, yet its performance degrades in tall arbor-type Pu-erh tea trees due to structural variations and lighting interference.

In comparison, CenterNet—an anchor-free framework relying on geometric centers and keypoints for object detection—often struggles with fine-grained detection tasks. This is primarily because its keypoint-based representation lacks explicit boundary regression and spatial context modeling, making it less effective at distinguishing densely distributed Pu-erh tea buds. In addition, its heavy backbone and large parameter size further limit its robustness and runtime efficiency in visually cluttered field environments.

To address these issues, the proposed ECS-Tea model introduces targeted architectural optimizations by integrating multi-scale spatial enhancement and channel attention mechanisms within a lightweight framework. This enables ECS-Tea to achieve superior accuracy while maintaining high robustness and real-time performance under complex environments. The radar chart clearly demonstrates that ECS-Tea significantly outperforms other models in terms of multi-metric balance, underscoring the effectiveness of its structural optimization design.

### 3.2 Visualization and analysis

**Figure 9:**
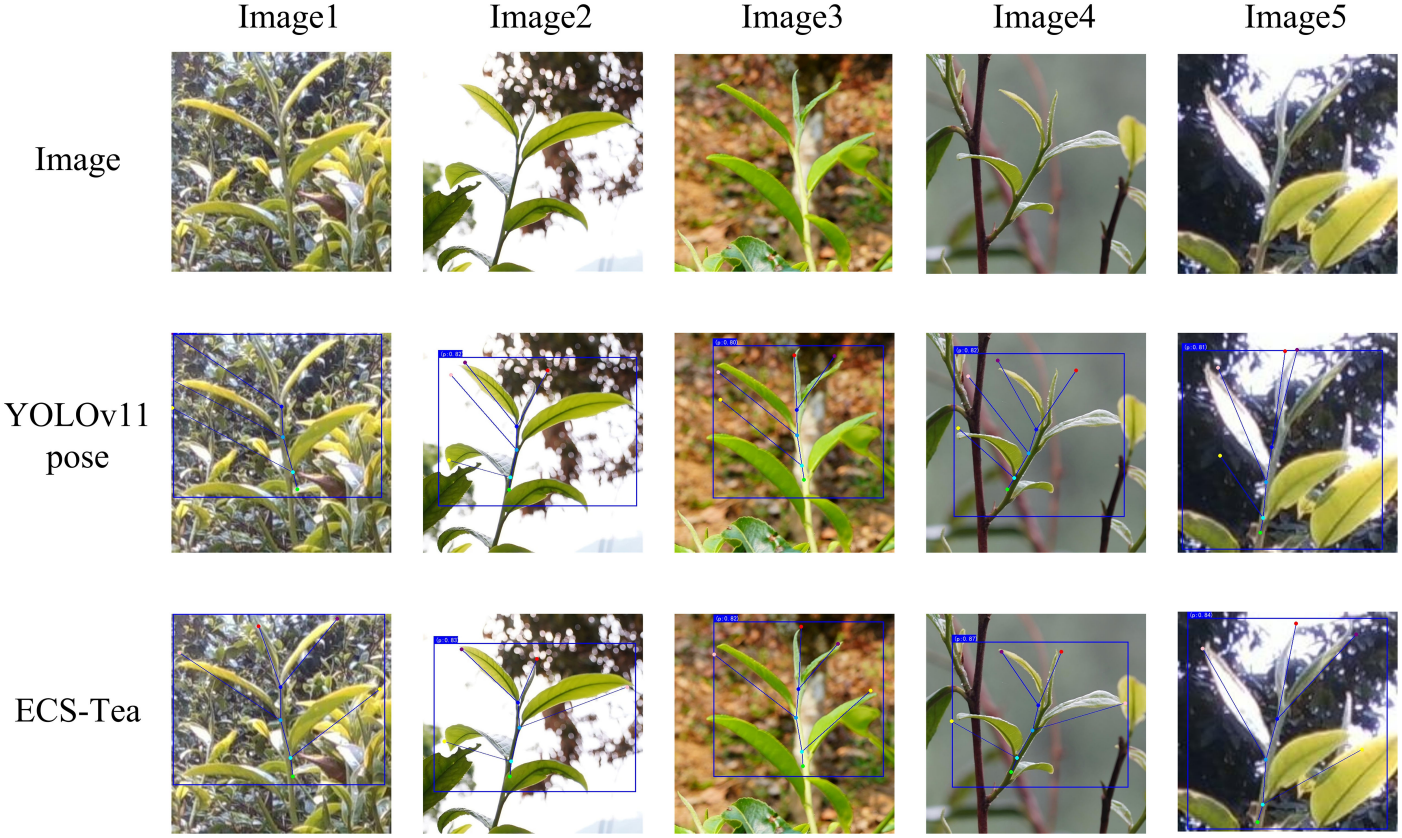
Single target recognition and key point detection results.

**Figure 10:**
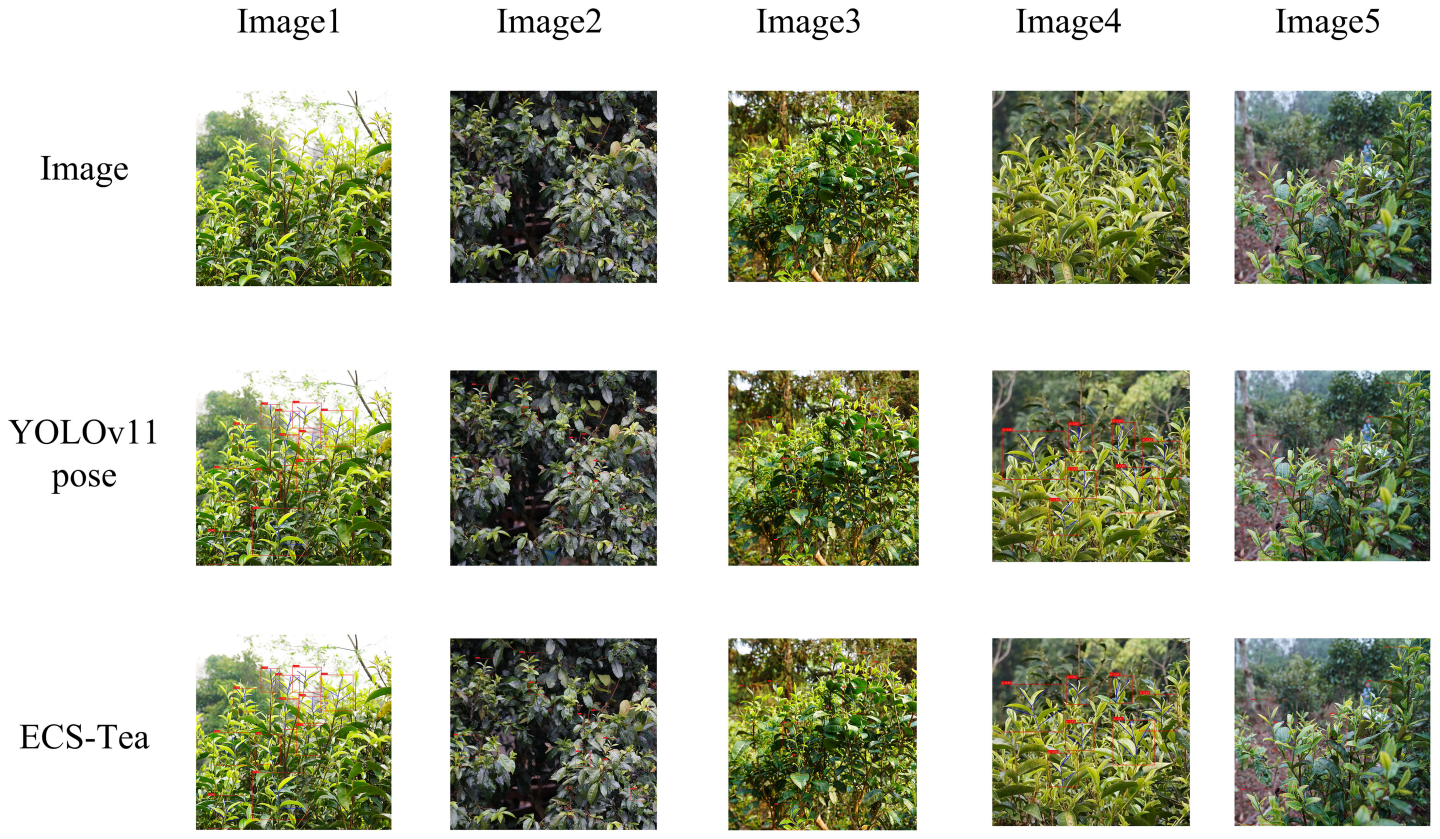
Results of multi-target recognition and key point detection.

**Figure 11:**
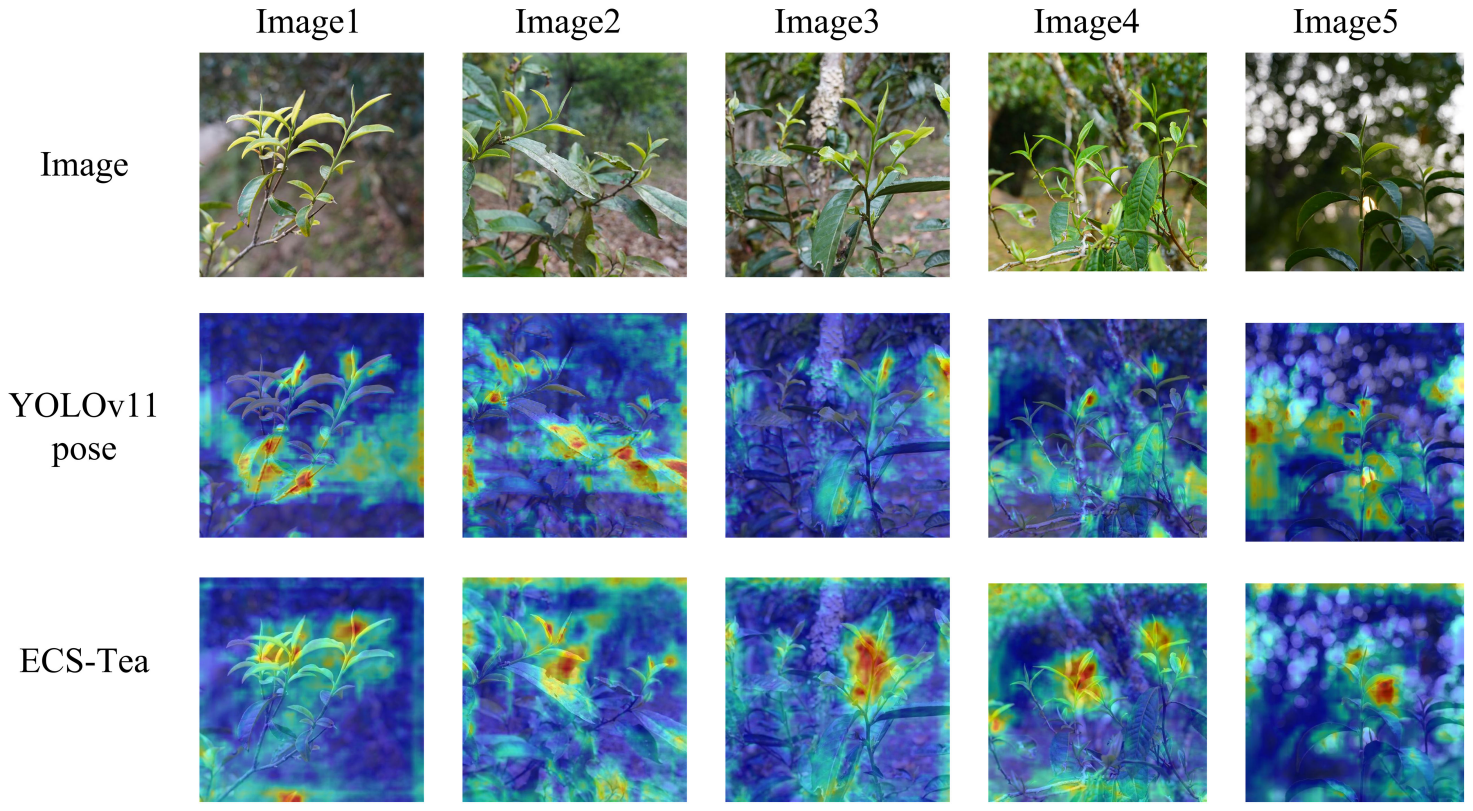
Overall Grad-CAM comparison results.

**Figure 12:**
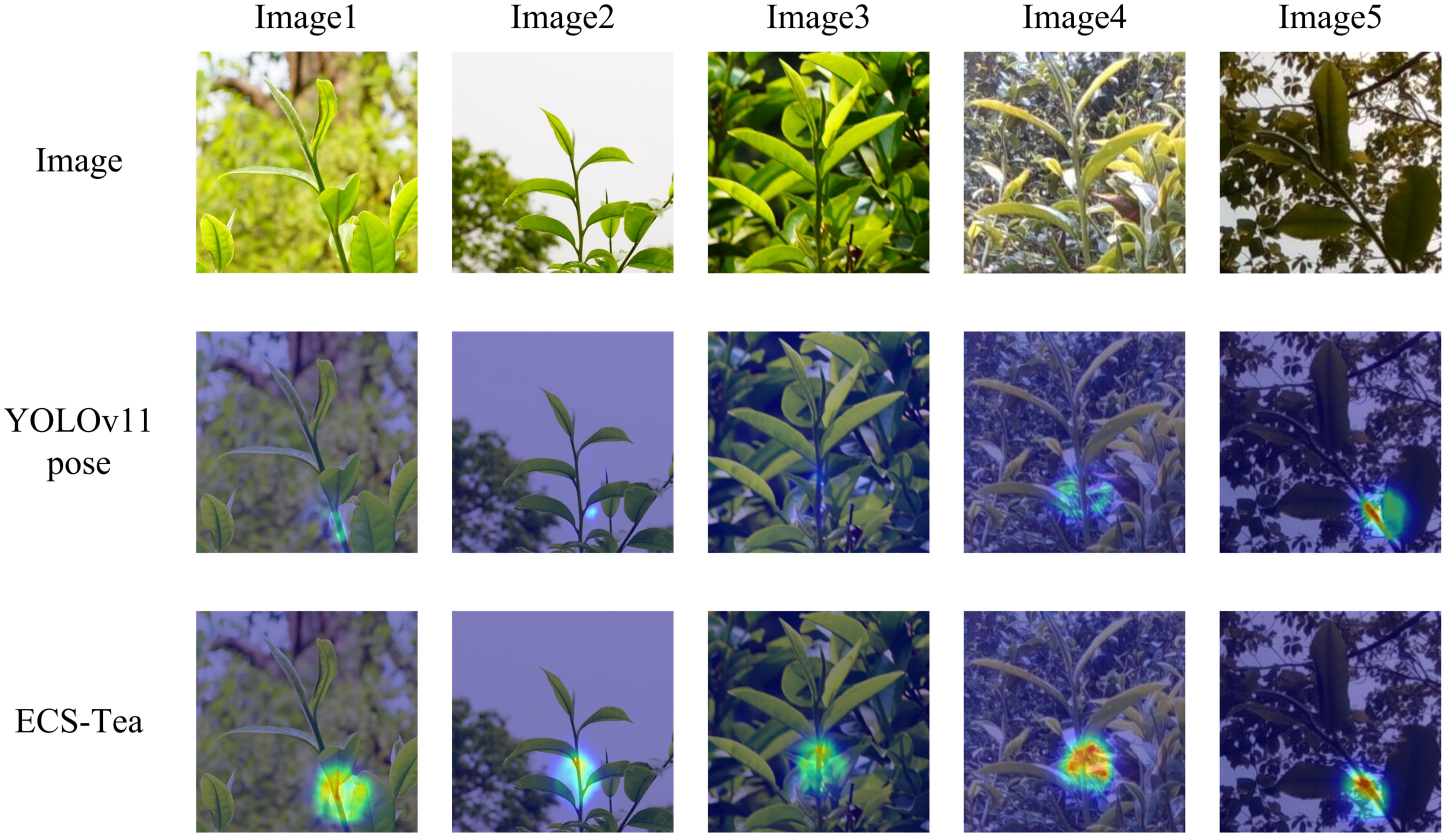
Comparison results of Grad-CAM at the picking point.

To further validate the proposed model’s adaptability and generalization ability in real-world scenarios, and to systematically analyze its advantages in feature extraction and decision reasoning, this study conducted visualization experiments using an independent external test dataset. The external dataset consisted of Pu’er tea shoot images collected at different time periods and under diverse illumination conditions (e.g., natural light variation, partial shadow, and backlighting), ensuring that the evaluation was independent of the training and validation data. This study selected four representative scenarios at different visual levels and task dimensions for visualization analysis: Single target recognition and key point detection results (**Figure 9**), Results of multi-target recognition and key point detection (**Figure 10**), Overall Grad-CAM comparison results (**Figure 11**), and Comparison results of Grad-CAM at the picking point (**Figure 12**). Among them, the single-plant and multi-plant detection results were used to evaluate the model’s detection accuracy and keypoint consistency under different target densities and background complexities; the overall Grad-CAM visualizations were used to show the distribution of feature attention at the global semantic level; and the picking-point Grad-CAM visualizations focused on the model’s fine-grained perception and localization ability in operational regions (Santos et al., 2025). Through the above multidimensional visualization comparisons, the performance advantages of the ECS-Tea model in detection accuracy, feature representation, and attention distribution can be comprehensively revealed.

**Figure 9** illustrates the visualized results of single-plant tea bud detection and keypoint localization. The proposed ECS-Tea model maintains accurate boundary delineation even under challenging conditions such as complex illumination, cluttered backgrounds, and partial occlusions. Its keypoint distribution exhibits higher precision and spatial coherence, highlighting enhanced robustness and stable localization performance across varied environments. In contrast, the baseline YOLOv11-Pose model suffers from blurred boundaries, incomplete part recognition, and keypoint drift under complex scene conditions. These issues are particularly pronounced in terminal and occluded regions, suggesting that its spatial attention distribution is more dispersed, thereby hindering consistent structural prediction and reducing reliability in fine-grained localization tasks.

**Figure 10** illustrates the detection performance under dense multi-plant distributions and structurally complex environments of Pu-erh tea buds. As the scene complexity increases, the baseline YOLOv11-Pose model exhibits noticeable missed and false detections, particularly in regions with overlapping targets or similar texture patterns, where keypoint predictions deviate from their true structural positions—revealing its limited ability to discriminate between multiple targets in cluttered visual scenes. In contrast, the ECS-Tea model effectively distinguishes adjacent targets and preserves structural and keypoint consistency under the same complex conditions. This improvement stems from the integration of Cross-Scale Feature Fusion (CSFF) and Spatial Self-Calibration Attention (SCSA) modules, which enhance contextual feature modeling, suppress background noise, and strengthen discriminative learning. Consequently, ECS-Tea achieves stable detection and high-precision localization even in high-density and high-noise agricultural scenes.

**Figure 11** depicts a Grad-CAM heatmap comparison between the two models across the entire image. The ECS-Tea model demonstrates tightly focused attention on the target body with smooth boundary transitions, suggesting that it more effectively captures high-level semantic representations and achieves clearer separation between foreground and background regions. In contrast, the YOLOv11-Pose model exhibits a dispersed and unfocused attention distribution, with misaligned activations frequently occurring around branch–leaf intersections or background clutter. This behavior reveals an overreliance on low-level texture cues, resulting in incomplete target perception and spatial localization deviations, particularly in visually complex environments.

**Figure 12** highlights the Grad-CAM heatmaps centered on picking keypoint regions, aimed at assessing the model’s fine-grained perceptual capacity in operationally critical areas. The results reveal that the ECS-Tea model produces highly concentrated and strongly activated responses around picking points, suggesting enhanced geometric sensitivity to structural details and superior semantic alignment in the representation of key regions. By contrast, the baseline YOLOv11-Pose model displays a diffuse and misaligned attention distribution, often focusing on irrelevant regions such as leaves or branches, implying limited perceptual sensitivity to actual operational targets and weaker structural discrimination. This distinction further underscores that the ECS-Tea model excels not only in macro-scale object recognition but also in micro-scale keypoint comprehension, achieving greater precision and temporal stability. Such performance establishes a robust perceptual foundation for subsequent automated picking and manipulation tasks.

In summary, the visual analyses provide compelling evidence that the ECS-Tea model consistently outperforms the baseline across single- and multi-target scenarios, demonstrating superior feature extraction, decision reliability, and structural awareness. By reducing missed detections, keypoint drift, and boundary ambiguity, ECS-Tea achieves holistic gains in precision, robustness, and interpretability, thereby offering a solid perceptual backbone for stable and explainable agricultural automation systems.

### 3.3 Spatial positioning performance experiment of Pu-erh tea tender shoot picking point

**Figure 13:**
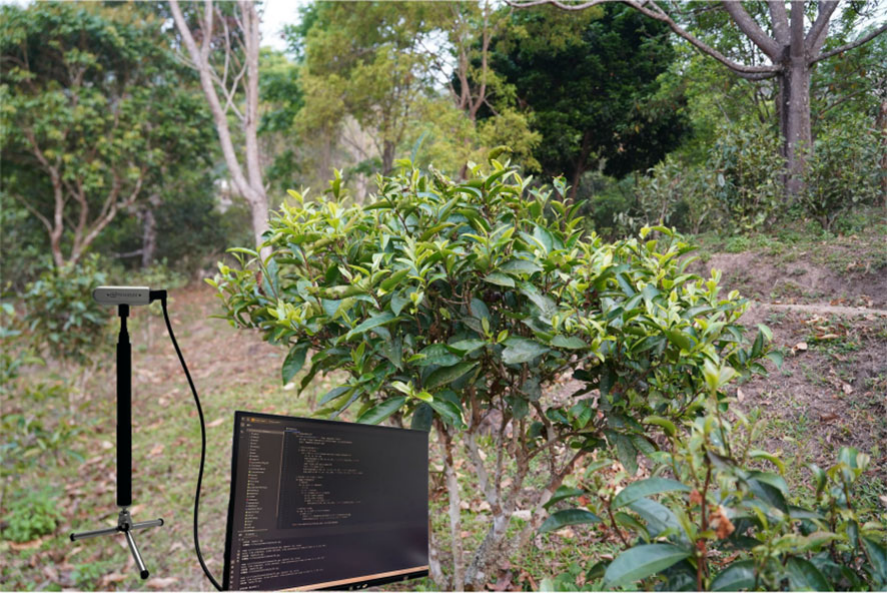
Schematic diagram of the positioning experiment.

To assess the effectiveness of the proposed multi-frame depth fusion method for 3D localization of Pu-erh tea tender shoot picking points, a localization and detection platform for Pu-erh tea tender shoots was established, as shown in **Figure 13**. The experiment employed the pyrealsense2 library as the Python interface to the Intel RealSense SDK to access the depth camera and employed OpenCV’s drawing function cv2.circle() to mark keypoint positions with circles in the image, and employed cv2.putText() to display the 3D coordinates (in millimeters) adjacent to the keypoints as text. To ensure high-precision benchmarking, the ground-truth 3D coordinates were obtained under controlled stable conditions using a BOSCH GLM30–23 laser rangefinder, which provides a measurement accuracy of ±1.5 mm, thereby establishing a reliable standard for quantitative evaluation.

**Figure 14:**
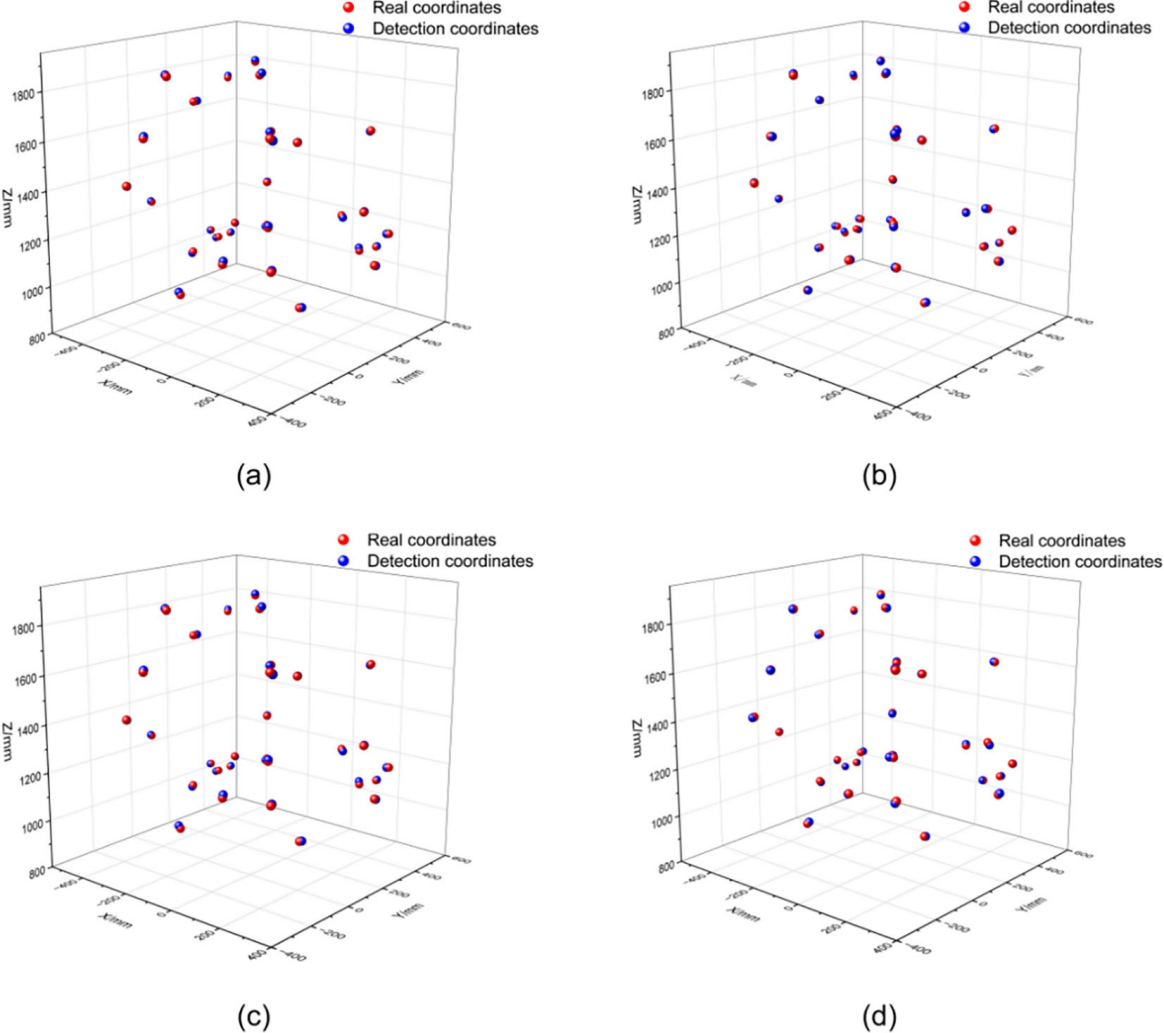
Schematic diagram of the positioning experiment **(a)** Single-frame depth estimation; **(b)** Multi-frame fusion (mean); **(c)** Multi-frame fusion (mode); **(d)** Adaptive fusion strategy.

A set of comparative experiments was designed to evaluate 3D keypoint localization error and the system’s robustness under four different strategies, with the 3D spatial localization results under a fixed ground-truth coordinate set presented as shown in **Figure 14**.

**Figure 14** shows that detection coordinates generated by the single-frame depth estimation method exhibit pronounced spatial deviations, resulting in a dispersed overall distribution. With the application of fusion strategies, the detection results progressively align with the ground-truth coordinates, leading to substantial improvement in spatial alignment. This effect is particularly evident under the adaptive fusion strategy, where the detected point cloud tightly conforms to the true distribution, yielding a notable improvement in spatial localization consistency.

**Figure 15:**
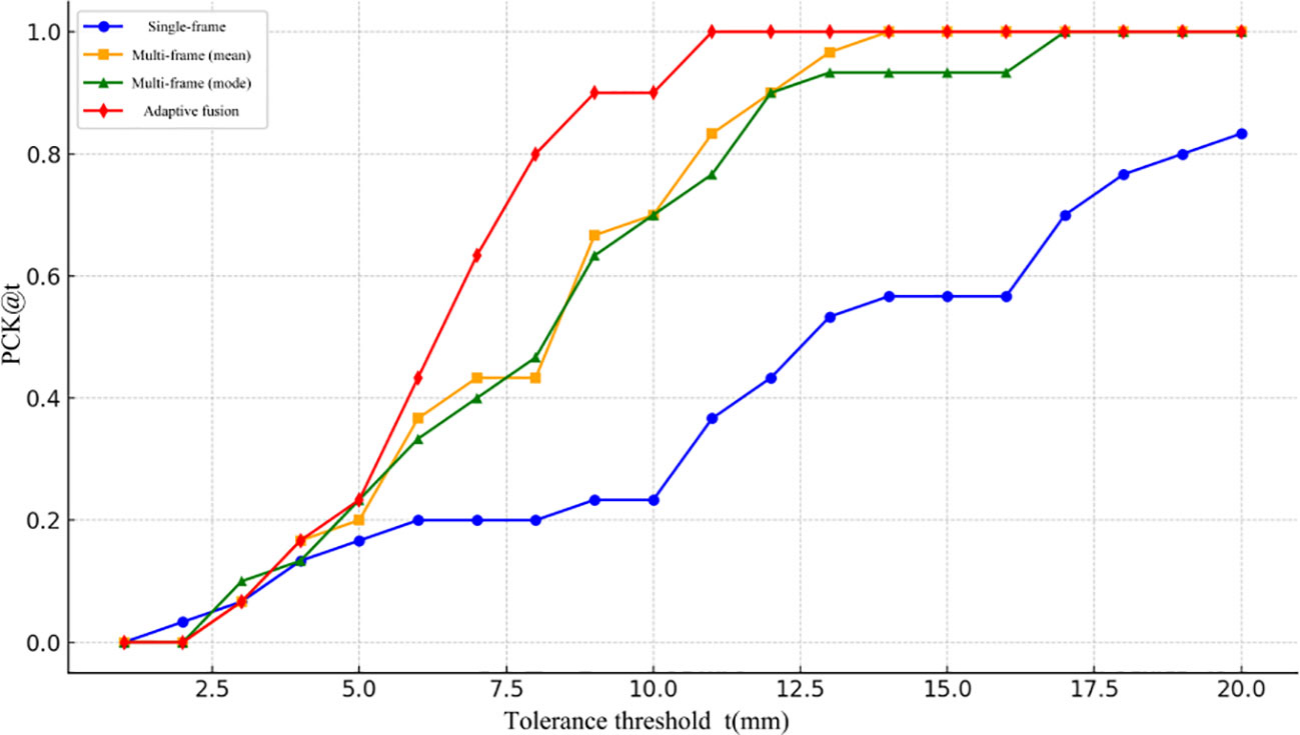
Comparison of accuracy of PCK@t with threshold for each method.

To more precisely assess the accuracy of different 3D localization approaches under varying error tolerance thresholds, this study plotted the PCK (Percentage of Correct Keypoints) curves, as illustrated in **Figure 15**. The x-axis represents the error threshold (t, in mm), while the y-axis indicates the proportion of keypoints whose prediction error falls below that threshold, namely PCK@t. To enhance the curve’s resolution and smoothness, the threshold interval was set to 0.5 mm, meaning one PCK value was sampled for every 0.5 mm increment. As shown, the proposed adaptive multi-frame fusion strategy consistently achieves superior localization accuracy across all threshold levels, demonstrating its robustness and applicability in real-world tea-picking scenarios.

**Figure 16:**
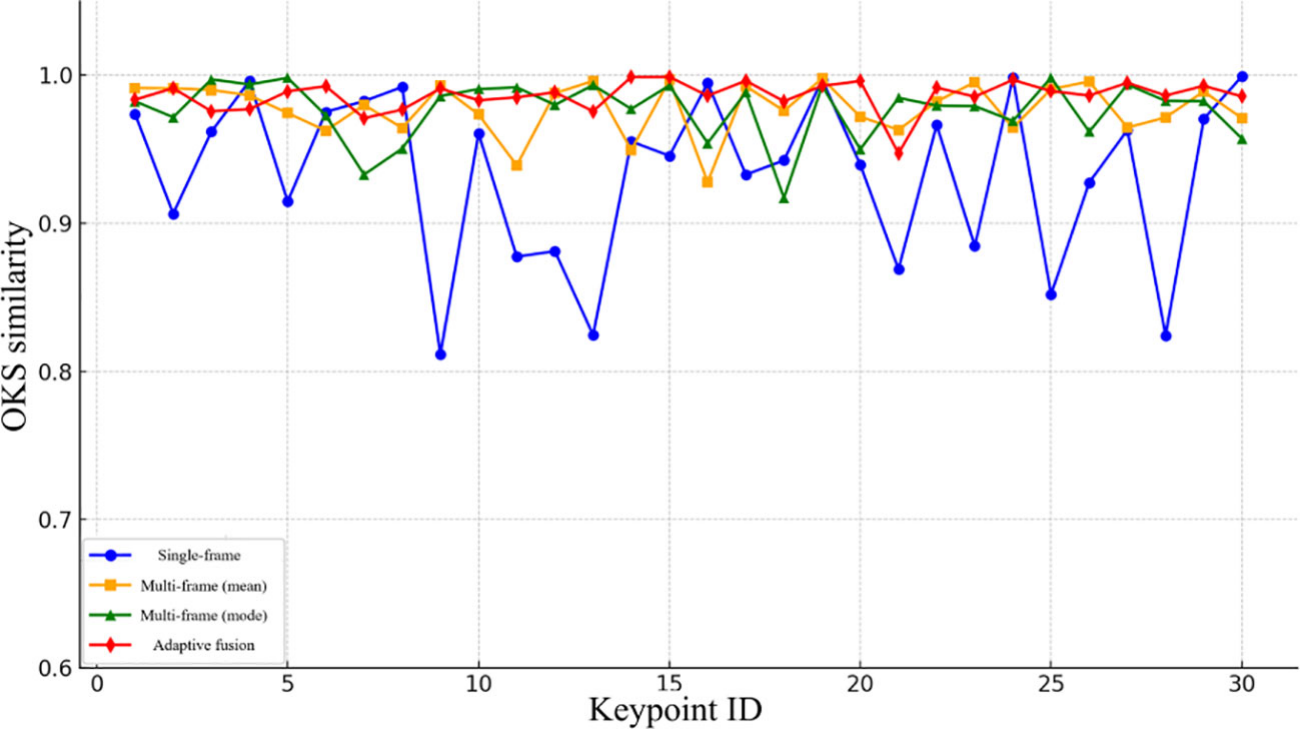
Comparison of OKS precision of different methods at each picking point.

To gain deeper insight into the stability of different localization methods across individual keypoints, **Figure 16** presents point-level evaluation results derived from the OKS (Object Keypoint Similarity) metric. As illustrated, the proposed adaptive fusion strategy consistently maintains high OKS values across the majority of picking points, underscoring its superior localization accuracy and robustness. In contrast, the single-frame depth estimation approach exhibits pronounced error fluctuations across multiple positions, indicating limited consistency. Although fusion-based methods (mean and median) perform better overall than single-frame estimation, they still exhibit localized accuracy degradation. These findings further confirm the effectiveness of the OKS metric in evaluating 3D localization precision and reinforce the advantages of the proposed adaptive fusion approach.

## 4 Conclusion

This paper presents ECS-Tea, a lightweight keypoint detection framework tailored for tea-picking applications, which integrates bionic structural keypoint annotation, an optimized EfficientNetV2 backbone, a CSFF-based cross-scale feature fusion mechanism, and an SCSA spatial self-calibration module, forming a multi-component architecture that achieves an optimal trade-off between accuracy and efficiency. Building upon this framework, an inter-frame keypoint tracking and adaptive multi-frame depth fusion strategy was introduced to enable temporally stable tracking of picking points in image sequences and high-precision estimation of their 3D spatial coordinates. Experimental results demonstrate that, compared with the YOLOv11Pose baseline, the ECS-Tea model achieves comprehensive performance improvements in keypoint detection: precision P(K) increased by 3.4%, recall R(K) by 3.8%, mAP@0.5(K) by 4.9%, and FPS by 11.1%, while model weight and parameter count were reduced by 49.1% and 49.2%, respectively. Regarding spatial localization performance, comparative evaluations using the PCK and OKS metrics reveal that ECS-Tea consistently achieves higher localization accuracy and structural coherence across various thresholds, substantially improving the stability and precision of 3D picking-point localization. In conclusion, the proposed ECS-Tea model delivers structured, high-precision, and temporally stable spatial information for intelligent tea-picking systems. It is well-suited for Pu-erh tea bud detection and localization under elevated operational conditions, effectively handling complex viewpoint variations, target occlusions, and leaf motion disturbances. The model demonstrates strong practical deployment potential in unstructured plantation environments and dynamic harvesting scenarios.

Despite the promising performance of this study in terms of accuracy, efficiency, and practical applicability, several limitations remain. The dataset was collected during a specific period and from a single tea cultivar, which may restrict the model’s cross-seasonal and cross-varietal generalization capability. Furthermore, keypoint annotations still depend on manual labeling, introducing subjectivity and time overhead. Future work may expand the dataset diversity and integrate semi-supervised learning frameworks together with multimodal sensing modalities (e.g., infrared imaging) to further enhance the model’s robustness and environmental adaptability, thereby offering stronger technical support for the real-world deployment of intelligent tea-picking systems.

## Data Availability

The datasets presented in this article are not readily available because the data may be used for future research. Requests to access the datasets should be directed to JCWang20000531@outlook.com.
